# The British Pasteur Institute

**Published:** 1891-06-20

**Authors:** 


### The Hospital

A Weekly Journal of

Science, Medicine, Nursing, and Philanthropy.

*For Hospitals, Asylums, Infirmaries, Dispensaries, Medical Practitioners, Students, Nurses, and the Charitable Public.*

Vol. X.—No. 247.] [June 20, 1891.

### THE BRITISH PASTEUR INSTITUTE.

The promoters of the British Institute of Preventive Medicine have applied to the President of the Board of Trade for incorporation under peculiar conditions; and the President, Sir Michael Hicks Beach, has refused their application. The applicants, it is to be remembered, consisted of men of undoubted scientific eminence, and of great personal weight. They claimed to represent, and they did in effect represent, the science and the medicine of this country. There have been opposed to them for some time certain persons of little or no scientific distinction, but of much personal weight in point of ability and character. The non-scientists, as they may be called, have carried the day against the scientists: an uninstructed humanitarianism has defeated in open conflict a humanitarianism which is instructed and specialised. It is now the duty of impartial judges, who may have knowledge of the questions at issue, to offer their testimony before the higher tribunal of unprejudiced public opinion.

The defeated scientists are angry; and since they are but men this is not unnatural. But it is necessary for them to bear in mind that science can no more advance without checks and obstructions, conflicts and martyrdoms, than can morals or religion, or even civilisation itself. Light, it is evident, is better and more beautiful than darkness; but no day is established without a previous struggle with night. A high morality is confessedly superior to crude and animal self-indulgence; but does not nine-tenths of the tragedy of life consist in the struggle to subdue the lower passions, and to live on the higher planes of conduct? The scientists feel a kind of scorn for their opponents, and express it freely. Therein they show that their studies in history and philosophy have been much less fruitful in their minds than their researches in the laboratory of material things. A greater magnanimity of mind would have displayed a nobler patience towards opponents who are in no sense unworthy.

Besides, these scientists must bear in mind that they have not yet made all their results so convincing that "he may run who reads." In the last resort public opinion will pronounce the final verdict on scientific facts and discoveries exactly as it pronounces the final verdict on politics, poetry, law, theology, and every other subject whatsoever. This is right; but whether it be right or not it is inevitable. Scientists must remember, too, and bacteriologists in particular, that they are not in the best possible odour at the present moment. The public has not yet forgiven Dr. Koch; and even if it had, the unprejudiced part of the medical profession has not forgiven him, and ought not to forgive him, except upon confession and restitution. Moreover, neither the public nor the medical profession can forget that certain scientists of great name and authority in this country rushed eagerly after the Koch delusion, and rent the air with their cheers over an undemonstrated hypothesis, which was afterwards demolished in less than six months. Do those scientists think that they can escape the consequences of their own acts? Do they for a moment imagine that either medical men or the public will accept them as infallible guides immediately after their performances at Berlin? If they do they must have exceedingly inconsequent minds; and they must hold the opinion that the minds of the medical profession and of the general public are equally inconsequent.

Those scientists who please themselves with the agreeable delusion of their own infallibility, and who imagine that the medical profession and the public take them at their own valuation, are simply making themselves a laughing-stock for all persons who are at the trouble to think. Kochism is dead, and cannot come to resurrection. Pasteurism, in so far as the word connotes Pasteur's anti-rabic treatment, is still an unproved hypothesis. Sir Joseph Lister, boldly affirming at the Board of Trade that Pasteur had been the means of saving twelve thousand persons from a horrible death by rabies, is an instance of child-like credulity which can only be matched by Cardinal Newman's devout belief in ecclesiastical miracles. Bacteriology is a science which promises wonderful practical fruits; but it must not be forgotten that that science is still in its earliest infancy. One of Sir Joseph Lister's pleas for the new institute was the necessity of making such extended investigations as were only possible at an institution of the kind proposed, in order that we might, sooner or later, demonstrate whether the tubercle bacillus be really the essential cause of consumption or not. But this confession of the crude state of bacterial knowledge generally is one of the strongest possible vindications of the position of those opponents who doubt the infallibility of Koch and Pasteur.

It seemed necessary to say something like what we have said, in justice to Sir Michael Hicks Beach, and those who have influenced him against the wishes of the scientists, and also in justice to the very considerable number of medical men who watch the bacteriological conflict with keen interest, but who refuse to commit themselves to a verdict upon insufficient evidence. Still, when all is said in defence of the anti-scientists which justice demands, no scientific medical man can approve of the obstructions and angry fightings against which science in these latter days has to contend. Freedom of thought and action is claimed for all men in this country, and may, therefore, be justly claimed for scientists. The scientific man has a conscience; and it is right that he should have the freedom to walk in the light of his own conscience rather than in that of another man's. No man or woman should feel at liberty to say to another, "I feel a certain course of action to be right and a certain other course to be wrong; and if any man feels differently from me, I shall do my utmost to prevent him acting according to his own judgment, and to compel him to act according to mine." This is the position which the anti-scientist takes up. He assumes at once that he is both wiser and kinder than the scientist. Such a position is not tenable for a moment by a really right-minded person. The only attitude of mind which a sound judgment can stand by is, that every man shall have the fullest right to the free exercise of his own judgment so long as the exercise of that judgment does not lead him to injure other human beings. The anti-scientists are not only obstructing and injuring the cause of science, but they are obstructing and injuring the cause of individual liberty and right. The scientist has a Divine Master exactly as the anti-scientist has; and it is not at the bar of the anti-scientist that he should be called upon to plead his cause, but at the bar of his own conscience. "To his own master he standeth or falleth."

That Britain owes it to itself to establish and maintain an Institute of Preventive Medicine is a proposition which does not admit of dispute. The science of the immediate future; the science which is destined to give us what may be nothing less than a new revelation; the science which may possibly be to our age what Judaism was to the age of Moses, and what Christianity was to the Roman Empire—this is the science which will be prosecuted at the Institute of Preventive Medicine. A noble Jew on a memorable occasion warned the leaders of his nation to avoid a conflict with Christianity, "lest, haply," he said, "ye be found even to fight against God." It is quite as possible to fight against God in the region of science as in the region of morals and religion. All the world is God's world, and all truth is God's truth. He is an atheist who would affirm otherwise. Sir Michael Hicks-Beach played an inconsequent part when he refused the application of the scientists. Those gentlemen must now go a step higher: they must appeal to Parliament and the nation, and they must appeal not only for countenance, but for support. Whilst we are the last to admit any man's claim to infallibility, be he scientist or be he Pope, we cannot but be aware of the fact that in this country, and at the present moment, there is a noble army of scientific workers which is worthy of any age or any country. What those scientists demand and must have is freedom. Let us judge them by results as severely as we will; but let us be ashamed even to think of putting on them such chains and manacles as will prevent them producing any results at all. Liberty of conscience, liberty of action, and all reasonable furtherance in his work is what every man has a right to insist upon, when his object is patriotic, his mind is sober, and his conscience is honest and true.

